# Classical Prognostic Factors Predict Prognosis Better than Inflammatory Indices in Locally Advanced Cervical Cancer: Results of a Comprehensive Observational Study including Tumor-, Patient-, and Treatment-Related Data (ESTHER Study)

**DOI:** 10.3390/jpm13081229

**Published:** 2023-08-03

**Authors:** Martina Ferioli, Anna Benini, Claudio Malizia, Ludovica Forlani, Federica Medici, Viola Laghi, Johnny Ma, Andrea Galuppi, Savino Cilla, Milly Buwenge, Gabriella Macchia, Claudio Zamagni, Luca Tagliaferri, Anna Myriam Perrone, Pierandrea De Iaco, Lidia Strigari, Alessio Giuseppe Morganti, Alessandra Arcelli

**Affiliations:** 1 Radiation Oncology, Department of Medical and Surgical Sciences-DIMEC, Alma Mater Studiorum University of Bologna, 40138 Bologna, Italy; anna.benini19@gmail.com (A.B.); ludovica.forlani@studio.unibo.it (L.F.); federica.medici4@studio.unibo.it (F.M.); viola.laghi@studio.unibo.it (V.L.); johnny.ma@studio.unibo.it (J.M.); mbuwenge@gmail.com (M.B.); myriam.perrone@aosp.bo.it (A.M.P.); pierandrea.deiaco@unibo.it (P.D.I.); alessio.morganti2@unibo.it (A.G.M.); alessandra.arcelli@aosp.bo.it (A.A.); 2 Radiation Oncology, IRCCS Azienda Ospedaliero-Universitaria di Bologna, 40138 Bologna, Italy; andrea.galuppi@aosp.bo.it; 3 Nuclear Medicine, IRCCS Azienda Ospedaliero-Universitaria di Bologna, 40138 Bologna, Italy; claudio.malizia@aosp.bo.it; 4 Medical Physics Unit, Gemelli Molise Hospital-Università Cattolica del Sacro Cuore, 86100 Campobasso, Italy; savinocilla@gmail.com; 5 Radiotherapy Unit, Gemelli Molise Hospital, Fondazione Policlinico Universitario A. Gemelli, IRCCS, 86100 Campobasso, Italy; gabriella.macchia@gemellimolise.it; 6 Addarii Medical Oncology Unit, IRCCS Azienda Ospedaliero-Universitaria di Bologna, 40138 Bologna, Italy; claudio.zamagni@aosp.bo.it; 7 Gemelli ART (Advanced Radiation Therapy)—Interventional Oncology Center (IOC), Fondazione Policlinico Universitario Agostino Gemelli, IRCCS, 00168 Roma, Italy; luca.tagliaferri@policlinicogemelli.it; 8 Division of Gynecologic Oncology, IRCCS Azienda Ospedaliero-Universitaria di Bologna, 40138 Bologna, Italy; 9 Medical Physics, IRCCS Azienda Ospedaliero-Universitaria di Bologna, 40138 Bologna, Italy; lidia.strigari@aosp.bo.it

**Keywords:** anemia, brachytherapy, chemoradiation, cervical cancer, hemoglobin, inflammatory index, nutritional index, overall survival, observational study, prognostic factor

## Abstract

Systemic inflammation indices were found to be correlated with therapeutic outcome in several cancers. This study retrospectively analyzes the predictive role of a broad range of systemic inflammatory markers in patients with locally advanced cervical cancer (LACC) including patient-, tumor-, and treatment-related potential prognostic factors. All patients underwent definitive chemoradiation and pretreatment values of several inflammatory indices (neutrophil/lymphocyte ratio (NLR), platelet/lymphocyte ratio, monocyte/lymphocyte ratio, systemic immune inflammation index (SII), leukocyte/lymphocyte ratio, combination of platelet count and NLR, aspartate aminotransferase/platelet ratio index, aspartate aminotransferase/lymphocyte ratio index, systemic inflammatory response index, and aspartate transaminase/neutrophil ratio index) were calculated. Their correlation with local control (LC), distant metastasis-free (DMFS), disease-free (DFS), and overall survival (OS) was analyzed. One hundred and seventy-three patients were included. At multivariable analysis significant correlations were recorded among clinical outcomes and older age, advanced FIGO stage, lower hemoglobin levels, larger tumor size, and higher body mass index values. The multivariate analysis showed only the significant correlation between higher SII values and lower DMFS rates (*p* < 0.01). Our analysis showed no significant correlation between indices and DSF or OS. Further studies are needed to clarify the role of inflammation indices as candidates for inclusion in predictive models in this clinical setting.

## 1. Introduction

Cervical cancer is one of the most common cancers worldwide [1]. Concurrent chemoradiation (CRT) is the standard treatment option for patients with locally advanced cervical cancer (LACC). Although CRT achieves high rates of local tumor control [2], about one third of patients show treatment failure after the treatment [3,4]. In the literature, several prognostic models, also for cervical cancer patients, have been published in last years. They could help clinicians in predict clinical outcomes allowing a more and more personalized treatment, based on stage, risk of recurrence, and demographic characteristics. Multiple predictors have been studied and included in predictive models and, in the LACC setting, tumor size, histological type, lymph node metastases, and FIGO stage are prognostic factors significantly related to overall survival (OS) [5,6]. Furthermore, anemia has been known for decades to be a negative prognostic factor in LACC patients [7,8,9,10]. However, among published predictive models there is often heterogeneity for clinical setting, analyzed outcomes and included predictors, which makes it sometimes difficult to apply the model in the real daily practice.

In order to improve the outcome prediction, and therefore to allow treatment modulation based on the prognostic profile, recent investigations evaluated the predictive role of several systemic inflammation indices which were found to be significantly correlated with the therapeutic outcome in several cancers [11]. In particular, increased neutrophil-lymphocyte ratio (NLR) and platelet–lymphocyte ratio (PLR) were found to be related to worse disease-free survival (DFS) and OS [12,13,14,15,16,17,18,19,20,21,22,23,24,25,26,27] as well as worse cancer response after CRT [25] in LACC patients.

However, most of these studies analyzed only one index or a limited number of indices with partial assessment of potential confounders. Therefore, the aim of this study was to analyze the predictive role of a broad range of pre-treatment nutritional and systemic inflammatory markers, in a large population of patients with LACC treated with standard CRT, including clinical prognostic factors such as clinical, nutritional, tumor-related, and treatment-related data. 

## 2. Materials and Methods 

### 2.1. Aim and Design of the Study 

The aim of this study was to correlate the prognostic impact in LACC of different pre-treatment nutritional and systemic inflammation indices on the following clinical endpoints: local control (LC), distant metastasis free survival (DMFS), DFS, and OS. We retrospectively analyzed patients treated in our institution from July 2007 to July 2021 and enrolled in an observational study approved by our local Ethical Committee (ESTHER study, code CE 973/2020/Oss/AOUBo). Patients signed an informed consent to participate in the study. No patients were excluded from our analysis, to make the correlation as much as possible corresponding to daily practice.

### 2.2. Staging, Treatment, and Follow-Up

LACCs were retrospectively classified according to the 2018 FIGO staging system [28]. Patients underwent definitive CRT using a combination of external beam radiotherapy (EBRT) to the pelvis (45–50 Gy, 1.8–2 Gy per fraction) and intracavitary interventional radiotherapy (brachytherapy-BRT, either with pulsed or high dose rate) to reach a total equivalent dose of 85–90 Gy on the macroscopic primary tumor. The clinical target volume (CTV) was defined as the gross tumor volume, the uterus, the upper third of the vagina, the parametria, and the pelvic nodes (internal, external, and common iliac, obturator, and presacral nodes) with a 7 mm expansion. Para-aortic lymph nodes were irradiated only in case of nodal metastases in this nodal region. The planning target volume was defined as the CTV plus 10 mm isotropic expansion. Suspicious or metastatic pelvic nodes received a sequential or simultaneously integrated boost up to a total equivalent dose of 55–65 Gy. A daily check of the patient set-up was performed by electronic portal imaging device until 2015 and subsequently by on-board cone-beam CT [29]. Concurrent chemotherapy consisted of intravenous Cisplatin (40 mg/m^2^ weekly). Patients were followed up with physical examination every three months for two years and then every six months for the next three years. A thoracic-abdominal-pelvic computed tomography (CT) was performed if clinically indicated or every six months in the first two years and every year in the following three years.

### 2.3. Evaluated Parameters

#### 2.3.1. Patients Related Data

The following data were included in this analysis: age, body mass index (BMI, calculated as weight (Kg) divided by the square of height (m)), hemoglobin level (Hg, in g/100 mL), and prognostic nutritional index (PNI, calculated as serum albumin multiplied by 10 (g/dL) + 0.005 × total lymphocyte count (per mm^3^)). All these data refer to before CRT started.

#### 2.3.2. Tumor Related Data

The following data were included in this analysis: histological type (squamous cell carcinoma, adenocarcinoma), Federation of Gynecology and Obstetrics (FIGO) stage, based on the 2018 version, clinical tumor stage, clinical nodal stage, and maximum tumor diameter. 

#### 2.3.3. Treatment Related Data

The following data were included: radiotherapy technique (3-D conformal radiotherapy, intensity modulated radiotherapy, or volumetric modulated arc therapy, EBRT dose (Gy) and fractionation on the pelvis, brachytherapy boost dose (Gy), total tumor dose (Gy), and overall treatment time (EBRT plus BRT, days).

#### 2.3.4. Inflammatory Indices

The following inflammatory indices were analyzed: NLR, PLR, monocyte-to-lymphocyte ratio (MLR), systemic immune inflammation index (SII, calculated as platelet × neutrophil/lymphocyte), leukocyte-to-lymphocyte ratio (LLR), combination of platelet (PLT) count and NLR (COP-NLR, scored as follows: 0: NLR < 3 and PLT < 300 × 10^9^/L; 1: NLR > 3 or PLT > 300 × 10^9^/L; 2: NLR > 3 and PLT > 300 × 10^9^/L), aspartate aminotransferase/platelet count ratio index (APRI, calculated as [aspartate aminotransferase {IU/L}/upper limit normal/PLT {×10^9^/L}] × 100), aspartate aminotransferase-to-lymphocyte ratio index (ALRI, calculated as aspartate aminotransferase value [U/L]/lymphocyte count [× 10^9^/L]), systemic inflammatory response index (SIRI, calculated as neutrophil × monocyte/lymphocyte), aspartate transaminase to neutrophil ratio index (ANRI, calculated as aspartate aminotransferase/neutrophils). As for collected patients′ data, all indices referred to routine blood exams performed before CRT.

### 2.4. Statistical Analysis

Patient and tumor characteristics and treatments data were reported using descriptive statistics. Categorical data were reported with numbers and percentages while continuous data were reported with medians and ranges. LC was calculated as the time since CRT start to local-regional recurrence, as evidenced by imaging studies or clinical findings, or until last follow-up in patients without pelvic recurrence. DMFS was calculated as the time since CRT start to distant failure, as evidenced by imaging studies or clinical findings, or until last follow-up in patients without extra-pelvic recurrence. DFS was calculated as the time since CRT start to any treatment failure, or until last follow-up in patients without LACC recurrence. OS was calculated as the period from CRT start until death or the date of the last follow-up. For each of the four considered endpoints, a univariate Cox′s regression was performed including all the variables specified above. Moreover, a multivariate Cox′s regression was performed including all variables showing a *p*-value less than 0.25 in univariate analysis. A 5% level of statistical significance was used (*p* < 0.05). In both univariate and multivariate analysis, the impact on the various endpoints of the inflammation indices was performed considering the latter as continuous variables, and therefore without dichotomizing them using prespecified cut-offs. We did the same with other continuous variables, such as age, BMI, and tumor diameter. Data were analyzed using SPSS for Windows (version 20.0; SPSS Inc., Chicago, IL, USA).

## 3. Results

### 3.1. Patients′ Characteristics

One hundred and seventy-three patients were included in this analysis. Patients′ characteristics are reported in Table 1. Median follow-up was 36 months (range: 3–151 months). 

### 3.2. Treatment Characteristics 

All patients underwent concurrent CRT with weekly Cisplatin. Treatment characteristics are shown in Table 1. Positive lymph nodes were treated in 57 patients with an additional dose delivered either with sequential or simultaneous boost. BRT was delivered in all patients as Pulsed or High Dose Rate BRT. In our retrospective analysis we included all LACC treated patients from July 2007 to July 2021 in our institution, also including those who have interrupted or modified the treatment, mainly for clinical reasons. In this regard, we would like to report a patient with a known psychiatric pathology who prematurely stopped her EBRT treatment (26 Gy), due to poor compliance, for which we then personalized the dose of BRT boost (42 Gy). Moreover, a patient with a very large and not uniform tumor, after the first BRT fraction (4 Gy), was boosted with EBRT because the tumor and organs a risk anatomy did not permit us to deliver an accurate and safe BRT treatment.

### 3.3. Clinical Outcomes

During the follow-up 30 patients showed a local-regional recurrence, while distant metastases were recorded in 42 patients. Overall, 60 patients showed a treatment failure and 42 patients died. Moreover, 2-year LC, DMFS, DFS, and OS was 83.0%, 79.9%, 69.1%, and 87.4%, respectively, and 5-year LC, DMFS, DFS, and OS was 82.1%, 74.7%, 64.0%, and 71.5%, respectively. Median LC, DMFS, and DFS was not reached, while median OS was 122 months (95%CI: 117-NR).

### 3.4. Prognostic Impact of the Analyzed Parameters 

#### 3.4.1. Patients and Treatment Related Data

Older patient age was significantly correlated with lower DMFS rates at both univariate and multivariate analyses. Similarly, older patients had lower OS rates at both univariate and multivariate analysis. Furthermore, higher BMI values were significantly correlated with worse DFS and worse OS, both in univariate and multivariate analysis. Moreover, Hb values >12 g/dL resulted (compared to patients with Hb < 10 g/dL) in better LC, better DFS, and higher OS rates. Finally, patients with Hb > 12 g/dL showed better DFS at multivariate analysis even compared to patients with Hb levels between 10 and 12 g/dL (Table 2).

As regards the clinical outcomes, compared to patients with FIGO stage I-II LACC, patients with FIGO stage III showed, at univariate analysis, worse results in terms of LC, DMFS, DFS, and OS. At multivariate analysis only negative correlations with DMFS, DFS and OS were confirmed. Furthermore, patients with FIGO stage IV, compared with stage I-II, showed worse LC and DFS (both: *p* < 0.01) at univariate analysis but these correlations were not confirmed at multivariate analysis. Finally, larger tumor diameter correlated with worse LC, DFS, and OS. Instead, multivariate analysis confirmed only the negative correlation with LC (Table 2).

Moreover, none of the treatment-related parameters was significantly correlated with any of the analyzed outcomes 

#### 3.4.2. Inflammatory Indices

Higher COP-NLR scores and higher ANRI values were significantly correlated with lower LC rates at univariate analysis, but these correlations were not confirmed at multivariate analysis. Higher SII values were significantly correlated with lower DMFS rates at both univariate and multivariate analysis, as well as lower DFS rates, only at univariate analysis. None of the analyzed indices showed significant correlations with OS. (Table 2)

## 4. Discussion

In a comprehensive analysis of inflammatory indices and patient-, tumor-, treatment-, and nutrition-related parameters, the negative prognostic impact of older age, advanced FIGO stage, lower hemoglobin levels, and largest tumor size was recorded in LACC patients treated with CRT plus BRT boost. These results have been known for a long time even if, at least with regard to age, the relationship with outcomes seems to have a complex J-shaped nonlinear correlation, with some studies showing a worse prognosis even in younger patients’ subgroups [13,27].

In terms of nutritional parameters, our study showed a negative effect of BMI on DFS and OS, as previous studies [30]. However, similarly to the correlation with age, the complex relationship between BMI and prognosis should be highlighted. In fact, not only high values but also lower than normal values (BMI < 18.5) seem to be associated with a worse prognosis [31,32]. Furthermore, also studies based on the analysis of sarcopenia gave conflicting results in this setting, with analyses showing a significantly unfavorable impact of this parameter on OS [33], and studies failing to demonstrate this effect [34,35]. Our analysis did not show an impact of PNI on any of the evaluated endpoints, contrary to what was recorded in a previous study [12]. These contradictory results could arise from the different methodologies of these two analyses. Indeed, in the study by Haraga et al., the impact of the PNI was analyzed in combination only with age, nodal metastasis, FIGO stage, histological type, maximum tumor size, and PLR, while in our study also BMI, anemia, multiple inflammatory indices, and treatment characteristics were also included. Moreover, Gangopadhyay reported a significant impact of PNI on complete response rate after CRT but correlation with survival outcomes was not analyzed in her study [27]. 

Contrary to the literature data [36], no impact of treatment-related parameters on any of the analyzed endpoints was recorded in our analysis. An explanation for this outcome could result from the relative homogeneity of the delivered CRT and BRT, prescribed in one single center by the same group of radiation oncologists.

In terms of inflammation indices, our multivariate analysis confirmed only a significant correlation between increasing SII values and worse DMFS, in contrast to other studies reporting a significant correlation between pretreatment indices values and DFS [13,18,19,22,37] and OS [16,17,18,19,20,22,24,26,37]. This difference can be explained by several reasons. In fact, our study included the largest number of potentially confounding factors in the analysis (Table 3). Furthermore, unlike other studies, we did not evaluate the indices using predefined cut-offs or cut-offs defined based on ROC curve analysis but considering their values as continuous variables. In fact, our aim was to screen several indices in order to identify those able to impact on prognosis, even considering multiple confounding factors.

However, it should be noted that also other studies did not observe a significant impact on survival outcomes of pre-treatment inflammation indices [11,14,15,23] or reported a significant correlation only with DFS but not with OS [13]. Also in this regard, methodological or sample size differences could explain these dissimilarities.

In our study we analyzed only pre-treatment nutritional and inflammation indices. The reason for this choice is that predictive models, in LACC patients, seem useful only before and not after CRT. In fact, attempts to improve outcomes with treatments following CRT (e.g., adjuvant systemic treatments) were not successful, as demonstrated by very recent publications [38,39]. However, it should be noted that some studies showed a significant prognostic impact of post-treatment inflammatory indices or pre–post-treatment changes, even without significant correlations with pre-treatment values [19,23]. The results of these studies could be useful to plan further trials testing post-CRT adjuvant systemic therapies only in patients′ subgroups with higher risk of treatment failure.

Our study has obvious limitations. The number of analyzed patients, although relatively large, at least for some subgroup analyses may be too small to identify significant differences. Furthermore, even though we had planned a comprehensive analysis, some known prognostic factors were found to be unavailable in our series. For example, the squamous cell carcinoma cell antigen (SCC), useful in monitoring during follow-up [40], but also able to predict prognosis [41], was not included in the analysis due to the small number of patients with available data. Furthermore, even if our aim was to provide a comprehensive analysis of the inflammation indices in LACC, some of the indices used in the literature were not considered, such as platelet-to-neutrophil ratio, monocyte-to-neutrophil ratio, platelet-to-white blood cell ratio, platelet-to-monocyte ratio, lymphocyte-to-monocyte ratio, eosinophil-to-lymphocyte ratio, and eosinophil-to-monocyte ratio [42]. Finally, even if all our patients were into the LACC category, it was still a rather inhomogeneous series since the FIGO stage ranged from IB to IVA. This issue could have further limited the possibility of detecting the prognostic effect of inflammation indices considering that the latter, on the basis of a meta-analysis [43], seems to vary according to tumor stage and patient age.

Even considering these limitations, based on our and other published studies, further analyses of the prognostic impact of inflammation indices in LACCs seem warranted, also considering their favorable cost–effectiveness ratio. However, from a clinical practice point of view, incorporating the assessment of inflammatory indices into LACC management could be beneficial but at the moment, given the variability of scientific evidence, it would also seem premature.

Therefore, based on the available reports, the most promising candidate for inclusion in predictive models seem to be the NLR, given the significant prognostic impact recorded in several analyses [16,17,18,19,20,24,37], even though not confirmed in others [15,23] and our study. Moreover, future studies should be directed to analyze the possible combined impact of multiple inflammation indices. Indeed, the analysis by Lee et al. [14] showed worse OS only in case of increased pretreatment values of both NLR and PLR.

Finally, further analyses are needed to correlate the values and variations in inflammatory indices with the biological and molecular characteristics of the tumor and in particular to understand how these markers may be related to tumor response to therapies, both locally and out-of-target.

## Figures and Tables

**Table 1:** Patients′ characteristics.

Patients *n*° (%)	173 (100%)
Median age (range), years	56 (27–85)
Histological type, number of patients (%)	
Squamous cell carcinoma	173 (85.0)
Adenocarcinoma	26 (15.0)
Federation of Gynecology and Obstetrics stage, number of patients (%)	
IB	1 (0.6)
IIA	3 (1.7)
IIB	73 (42.2)
IIIA	9 (5.2)
IIIB	3 (1.7)
IIIC1	39 (22.5)
IIIC2	22 (12.7)
IVA	23 (13.3)
Radiotherapy technique, number of patients (%)	
3-D conformal radiotherapy	87 (50.3)
Intensity modulated radiotherapy	66 (38.1)
Volumetric modulated arc therapy	20 (11.6)
Median radiotherapy dose (range), Gy	
Prophylactic pelvic nodes irradiation	46.0 (26.0–50.4)
Metastatic nodes	57.5 (52.5–61.0)
Brachytherapy boost	28.0 (4.0–42.0)

**Table 2:** Univariate and multivariable Cox′s analysis.

	LC				DMFS				DFS				OS			
	Univariate		Multivariable		Univariate		Multivariable		Univariate		Multivariable		Univariate		Multivariable	
	HR/95%CI	*p*:	HR/95%CI	*p*:	HR/95%CI	*p*:	HR/95%CI	*p*:	HR/95%CI	*p*:	HR/95%CI	*p*:	HR/95%CI	*p*:	HR/95%CI	*p*:
**Age**	0.98/0.95–1.02	0.23			1.03/1.01–1.09	0.03	1.03/1.00–1.06	0.02	1.02/0.98–1.04	0.76			1.04/1.02–1.08	0.02	1.04/1.01–1.07	<0.01
**BMI**	1.01/0.98–1.11	0.22			1.04/1.00–1.10	0.05			1.10/1.02–1.14	<0.01	1.05/1.01–1.10	0.03	1.10/1.4–1.13	0.02	1.07/1.01–1.13	0.02
**PNI**	1.00/0.97–1.02	0.81			0.99/0.97–1.05	0.68			1.03/0.98–1.06	0.98			0.99/0.96–1.05	0.43		
**FIGO (I-II)**	Rif.				Rif.				Rif.				Rif.			
**FIGO (III)**	2.55/1.04–6.27	0.04			2.51/1.25–5.06	<0.01	2.86/1.41–5.82	<0.01	2.14/1.19–3.85	0.01	1.96/1.07–3.57	0.03	2.66/1.33–5.34	<0.01	3.27/1.58–6.79	<0.01
**FIGO (IV)**	4.91/1.77–13.58	<0.01			2.47/0.96–6.31	0.06			3.24/1.56–6.74	<0.01			2.00/0.75–5.34	0.165		
**T diameter** **(maximum)**	1.02/1.01–1.04	<0.001	1.02/1.01–1.03	<0.01	1.02/0.99–1.04	0.22			1.03/1.01–1.05	<0.001			1.02/1.01–1.03	<0.01		
**RBC**	0.46/0.27–0.78	<0.01			1.11/0.64–2.12	0.68			0.78/0.51–1.27	0.28			0.63/0.39–1.01	0.06		
**Hb (<10)**	Rif.				Rif.				Rif.				Rif.			
**Hb (10–12)**	0.41/0.17–1.01	0.05			0.41/0.13–1.25	0.11			0.44/0.19–1.01	0.05	0.41/0.17–0.98	0.04	0.58/0.22–1.52	0.27		
**Hb (>12)**	0.11/0.04–0.28	<0.001	0.14/0.05–0.36	<0.001	0.52/0.21–1.35	0.18			0.32/0.15–0.67	<0.01	0.37/0.17–0.79	0.01	0.34/0.14–0.85	0.02	0.23/0.08–0.61	<0.01
**NLR**	1.02/0.98–1.12	0.26			1.01/0.97–1.12	0.37			1.06/0.99–1.12	0.18			1.00/0.94–1.10	0.89		
**PLR**	1.01/0.99–1.03	0.28			1.03/1.01–1.05	0.02			1.02/1.01–1.03	0.02			1.01/0.99–1.04	0.46		
**MLR**	1.30/0.49–3.51	0.60			1.42/0.52–3.51	0.53			1.22/0.53–2.78	0.67			0.62/0.14–2.74	0.52		
**SII**	0.99/0.99–1.03	0.15			1.01/1.01–1.02	<0.01	1.02/1.01–1.03	<0.01	1.03/1.01–1.04	<0.01			1.04/0.98–1.11	0.27		
**LLR**	1.00/0.98–1.10	0.19			1.00/0.98–1.07	0.21			1.04/0.99–1.10	0.09			1.00/0.94–1.08	0.82		
**COP-NLR (0)**	Rif.				Rif.				Rif.				Rif.			
**COP-NLR (1)**	1.11/0.44–2.77	0.81			0.79/0.40–1.58	0.51			0.69/0.38–1.23	0.21			0.57/0.28–1.16	0.12		
**COP-NLR (2)**	2.72/1.09–6.79	0.03			0.97/0.43–2.21	0.95			1.07/0.55–2.07	0.83			1.11/0.51–2.39	0.78		
**APRI**	0.23/0.01–7.70	0.42			0.75/0.19–3.11	0.69			0.81/0.39–1.71	0.59			0.85/0.31–2.43	0.76		
**ALRI**	0.99/0.96–1.01	0.77			1.01/0.99–1.03	0.46			1.03/0.98–1.06	0.67			0.99/0.95–1.04	0.50		
**SIRI**	0.99/0.96–1.02	0.37			0.99/0.98–1.02	0.52			0.99/0.98–1.03	0.36			0.99/0.96–1.04	0.31		
**ANRI**	0.79/0.64–0.98	0.02			1.01/0.92–1.09	0.73			0.99/0.90–1.11	0.81			0.97/0.85–1.12	0.68		

Legend: ALRI: aspartate aminotransferase to lymphocyte ratio index; ANRI: aspartate transaminase to neutrophil ratio index; APRI: aspartate aminotransferase/platelet count ratio index; BMI: body mass index; COP-NLR: combination of platelet count and neutrophil to lymphocyte ratio; DFS: disease free survival; DMFS: distant metastasis free survival; FIGO: International Federation of Gynecology and Obstetrics; Hb: hemoglobin; HR: hazard-ratio; LC: local control; LLR: leukocyte-to-lymphocyte ratio; MLR: monocyte to lymphocyte ratio; NLR: neutrophil to lymphocyte ratio; OS: overall survival; PLR: platelet to lymphocyte ratio; PNI: prognostic nutritional index; RBC: red blood cells; SII: systemic immune inflammation index; SIRI: systemic inflammatory response index; T: tumor.

**Table 3:** Comparison between the results of previous analyses and those of our series.

Author, Year	Evaluated Indexes	Cut-Off	Outcome Predictions	Confounders Considered
Lee et al., 2012 [16]	NLR	1.9	<OS if >NLR (pre-CRT)	age; histological type; FIGO; treatment
Mizunuma et al., 2015 [18]	NLR	2.5	<OS and <PFS if >NLR (pre-CRT)	age; histological type; FIGO; T size; N stage; treatment
Haraga et al., 2016 [12]	NLR	2.85	<OS and <PFS if <PNI; no impact of NLR and PLR (pre-CRT)	histological type; FIGO; T size; N stage; lymphovascular invasion
	PLR	172.5		
	PNI	48.5		
Li et al., 2016 [26]	LMR	5.28	>PFS and >OS if >LMR (pre-CRT)	age; histological type; N stage; HPV status
Onal et al., 2016 [19]	NLR	3.03	<OS, <PFS if >NLR; no impact of PLR (pre-CRT)	age; histological type; FIGO; T size; N stage
	PLR	133.0		
Wang et al., 2016 [20]	NLR	2	<OS if >NLR (pre-CRT)	age; histological type; FIGO; T size; N stage
Koulis et al., 2017 [10]	NLR	5	<PFS and <OS if Hb <11.5; no impact of NLR alone (pre-CRT)	age; anemia; histological type; FIGO; T size; N stage; treatment
		11.5		
Holub et al., 2018 [22]	NLR	3.8	>OS if >ELR; <PFS if >PLR or >SII (pre-CRT)	age; histological type; FIGO; HPV status
	PLR	210		
	SII	1000		
	ELR	0.07		
Jonska-Gymrec et al., 2018 [24]	NLR	1.6	<OS if >NLR; no impact of PLR (pre-CRT)	age; histological type; FIGO; T grade; N stage
	PLR	158		
	MLR	0.33		
Jeong et al., 2019 [13]	NLR	2.8	<PFS if >NLR; no impact on OS	age; histological type; T size; FIGO; treatment
Gangopadhyay et al., 2020 [27]	PNI	44.8	>CR rate if PNI > 44.8	age; histological type; FIGO
Kim et al., 2020 [23]	NLR	2.33	<PFS and OS if >ΔNLR; no impact of NLR, PLR, LMR (pre-CRT) and of ΔPLR, ΔLMR	age; histological type; FIGO
	PLR	136.6		
	LMR	4.17		
Lee et al., 2020 [15]	NLR	3.04	<DFS if >NLR, >ΔNLR, >ΔPLR (post-CRT); <OS if >NLR (post-CRT); no impact on OS of NLR, MLR, PLR (pre-CRT), ΔNLR, ΔMLR, ΔPLR, and MLR, PLR (post-CRT)	age; histological type; FIGO; T size; N stage
	MLR	174.3		
	PLR	3.85		
Lee et al., 2021 [14]	NLR	2.34	<OS only if both >NLR and >PLR	age; histological type; FIGO; T size; N stage
	PLR	148.9		
Li et al., 2021 [17]	NLR	2.49	<OS and <PFS if >NLR and >MLR (pre-CRT); no impact of PLR, BLR, SIRI (pre-CRT)	age; histological type; T size; N stage; menopausal status
	PLR	154.2		
	MLR	0.26		
	SIRI	1.02		
	BLR	0.02		
Chauan et al., 2022 [25]	NLR	3	>CR rate if <NLR and <PLR	age; histological type; FIGO
	PLR	70		
Liang et al., 2022 [37]	NLR	3.87	<OSand <PFS if >NLR (pre-CRT)	age; BMI; histological type; FIGO; T size; N stage; treatment
Present series	NLR, PLR, MLR, SII, LLR, APRI, ALRI, SIRI, ANRICOP	c.v.	<distant metastasis-free survival if >SII	age; BMI; anemia; histological type; FIGO; T size; N stage; treatment; PNI
		0: NLR < 3 & PLT < 300;		
		1: NLR > 3 or PLT > 300;		
		2: NLR > 3 and PLT > 300.		

Legend: ALRI: aspartate aminotransferase to lymphocyte ratio index; ANRI: aspartate transaminase to neutrophil ratio index; APRI: aspartate aminotransferase/platelet count ratio index; BLR: basophil/lymphocyte ratio; BMI: body mass index; cN^+^: clinical positive nodes; COP-NLR: combination of platelet count and neutrophil to lymphocyte ratio; CR: complete response; CRT: chemoradiation; DFS: disease free survival; ELR: eosinophils lymphocyte ratio; FIGO: International Federation of Gynecology and Obstetrics; Hb: hemoglobin; LLR: leukocyte-to-lymphocyte ratio; MLR: monocyte to lymphocyte ratio; N: nodal; NLR: neutrophil to lymphocyte ratio; OS: overall survival; PFS: progression free survival; PLR: platelet to lymphocyte ratio; PNI: prognostic nutritional index; RT: radiotherapy; SII: systemic immune inflammation index; SIRI: systemic inflammatory response index; T: tumor.

## Data Availability

Data supporting the reported results will be made available on reasonable request.
